# Reply to the Letter to the Editor. Endoleak after endovascular aortic repair and lumbar vertebral erosion

**DOI:** 10.1007/s10195-015-0336-0

**Published:** 2015-02-26

**Authors:** Federico Mancini, Andrea Ascoli-Marchetti, Luca Garro, Roberto Caterini

**Affiliations:** University of Rome “Tor Vergata”, Rome, Italy

**Fig. 1:**
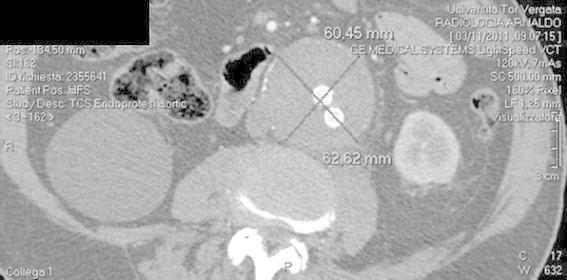
CT scan performed after EVAR procedure. The aneurysm was >6 cm in diameter

**Fig. 2:**
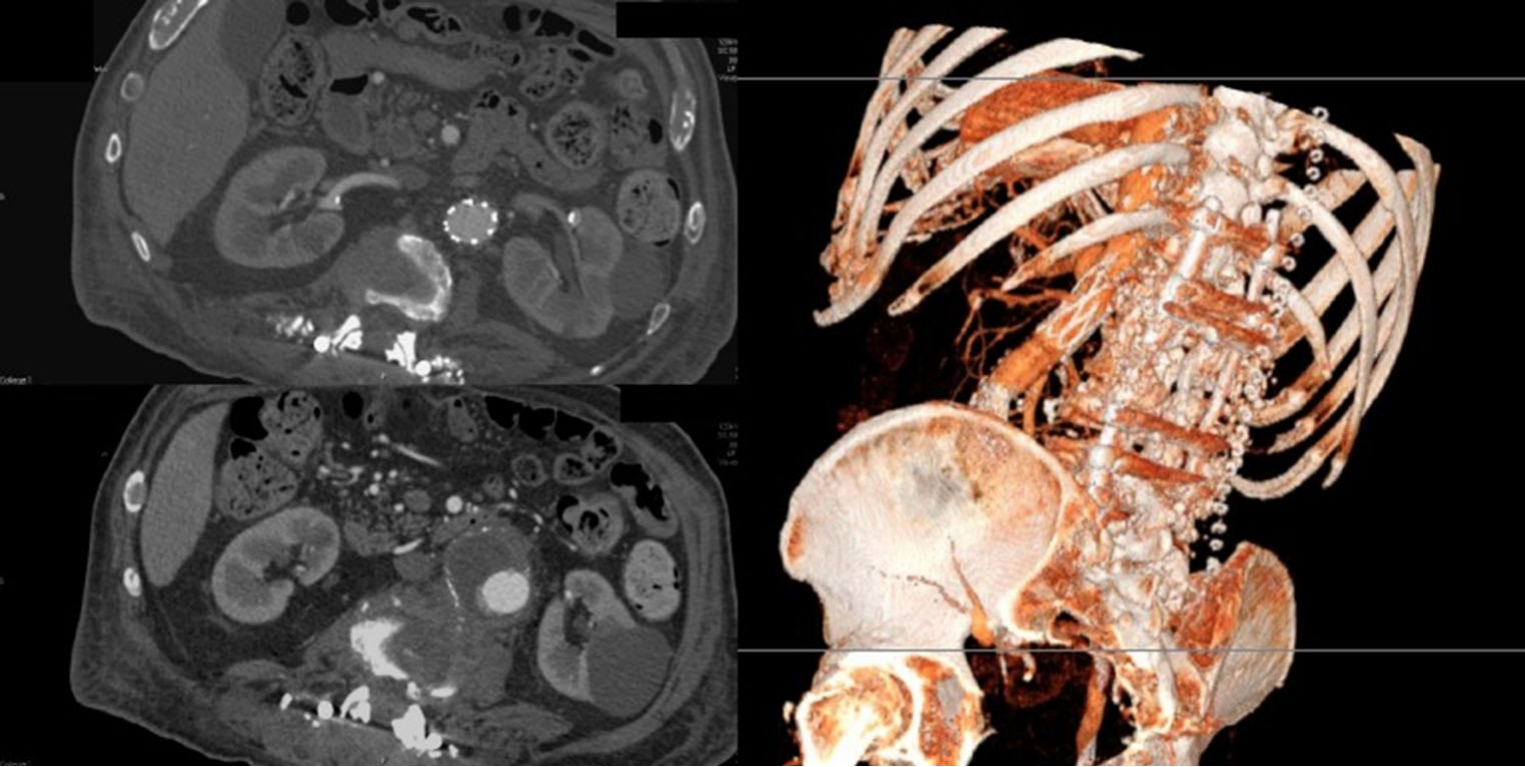
CT scan performed after spinal surgery showing the absence of infra/suprarenal endoleak

We thank Bozzani and co-workers for their comments [1] regarding this case [2]. It has been emphasized that this type of endoprosthesis does not generally promote rupture. In fact, in the most recent international trial, the incidence of type II endoleak (i.e., abnormal persistent post-endovascular aneurysm repair (EVAR) filling of the aneurysm sac through one or more of the branch vessels) after infrarenal implantation of a polymer-filled endovascular prosthesis was reported to be 34 % [3]; however, a significant increase in the diameter was documented in only one case (0.7 %). Moreover, ruptures did not result at 12-month follow-up but further observation has been recommended [4]. However, in this case the delay between implantation and the occurrence of an aneurysm was less than 1 year. We agree that in most cases the tamponade aortic rupture is determined by an endoleak or endotension. In this case, however, the aneurysm was extremely large (6.2-cm in diameter, Fig. 1), and this condition has been documented as a risk factor for rupture, including in well-positioned vascular endoprosthesis [5]. A computed tomography (CT) scan performed after the spinal procedure did not reveal any signs of an endoleak or bleeding (Fig. 2).
